# Low- versus High-Glycemic Index Mediterranean-Style Eating Patterns Improved Some Domains of Health-Related Quality of Life but Not Sleep in Adults at Risk for Type 2 Diabetes: The MEDGICarb Randomized Controlled Trial

**DOI:** 10.1016/j.tjnut.2024.07.005

**Published:** 2024-07-14

**Authors:** Anna Hjort, Robert E Bergia, Marilena Vitale, Giuseppina Costabile, Rosalba Giacco, Gabriele Riccardi, Wayne W Campbell, Rikard Landberg

**Affiliations:** 1 Division of Food and Nutrition Science, Department of Life Sciences, Chalmers University of Technology, Sweden; 2 Department of Nutrition Science, Purdue University, IN, United States; 3 Department of Clinical Medicine and Surgery, Diabetes, Nutrition and Metabolism Unit, Federico II University, Italy; 4 Institute of Food Sciences, National Research Council, Avellino, Italy

**Keywords:** glycemic index, Mediterranean diet, well-being, sleep, glycemic control

## Abstract

**Background:**

A healthy eating pattern such as the Mediterranean-style healthy eating pattern (MED-HEP) is associated with favorable effects on both cardiometabolic risk markers and self-reported health outcomes. Limited evidence exists regarding the influence of the glycemic index (GI) of carbohydrate foods consumed within a healthy eating pattern on self-reported health status and sleep.

**Objectives:**

To investigate the effects of a low- compared with high-GI MED-HEP on changes in health-related quality of life (HRQoL) and sleep.

**Methods:**

The MEDGICarb-intervention trial is a 12-wk randomized, controlled, parallel multi-center trial in adults with ≥2 features of the metabolic syndrome. Participants consumed an eu-energetic diet profiled as a MED-HEP with either low GI (experimental) or high GI (control). HRQoL and sleep were measured with Medical Outcomes Study 36-item short-form health survey version 2, Pittsburgh sleep quality index, and Epworth Sleepiness Scale at baseline and postintervention.

**Results:**

One hundred and sixty adults with ≥2 features of the metabolic syndrome completed the intervention [53% females, age 56 ± 10 y, body mass index (kg/m^2^) 31.0 ± 3.1]. Low- compared with high-GI MED-HEP resulted in differential changes between the groups in the HRQoL domains role physical [5.6 ± 2.2 arbitrary units (AU) compared with –2.5 ± 2.5 AU) and vitality (6.9 ± 1.7 AU compared with 0.0 ± 1.8 AU] (*P* < 0.05), which were driven mostly by improvements in the low-GI group. There were no significant differences between the MED-HEPs for changes in aggregated physical or mental components or for the other individual domains of HRQoL (physical functioning, bodily pain, general health, social functioning, role emotional, and mental health) or for sleep quality or daytime sleepiness.

**Conclusions:**

Low compared to high GI in the context of a MED-HEP resulted in modest improvements in some, but not all, health domains of HRQoL. No major differences were seen between the groups for measures of sleep.

This trial was registered at clinicaltrials.gov as NCT03410719.

## Introduction

Health-related quality of life (HRQoL) is a self-assessed measure of health that relates both to the participant’s burden of chronic diseases as well as the physical and mental aspects of well-being [1]. The HRQoL has been suggested as an important measure of self-perceived health to evaluate the subjective treatment effect in interventions and has also been shown to be a powerful predictor of morbidity and mortality [[2], [3], [4], [5]]. A growing body of evidence suggests an association between low HRQoL and greater cardiometabolic risk, including when the disease is not yet fully developed [6,7]. One of the most common tools to measure HRQoL is the short-form health survey (SF-36), which was developed from the Medical Outcomes Study (MOS). This is a 36-item questionnaire that captures 8 different aspects of physical health and mental well-being [8]. The MOS covered 40 dimensions of health in total, and the 8 chosen in SF-36 represent the most frequently measured in commonly used health surveys [8]. These are also those aspects of health that are considered most affected by disease and treatment [8]. Sleep is another important lifestyle factor that is related both to HRQoL [[9], [10], [11]] and to cardiometabolic health [[12], [13], [14]], where different aspects such as sleep duration, sleep quality, and daytime sleepiness can be considered.

During the last decade, the relationship between diet quality and self-reported health outcomes has gained more attention as several observational studies have shown associations between adherence to a healthy eating pattern (HEP) and subjective measures of health and well-being. A Mediterranean-style HEP (MED-HEP) represents 1 such eating pattern with well-established positive effects on cardiometabolic risk markers [[15], [16], [17], [18], [19]]. Moreover, several studies have been conducted suggesting associations between self-reported adherence to MED-HEP and better HRQoL [[20], [21], [22], [23]] and better sleep [[24], [25], [26], [27]]. However, most findings are from cross-sectional studies, and therefore, intervention studies investigating the treatment effect on such parameters are highly warranted.

Globally, cereals constitute the main source of total energy, plant-based protein, and dietary fiber in the diet. Whether cereals are consumed as refined grains or whole grains has large implications on the overall carbohydrate quality of the diet, which is of major importance for human health. The glycemic index (GI) of the food is 1 aspect of carbohydrate quality, which is a food trait of relevance for the postprandial glucose response of the meal. Loss of postprandial glucose control is often detected before an impairment in fasting glucose and has been proposed as an early step in the development of type 2 diabetes mellitus [28]. Glycemic variability is a marker of fluctuations in blood glucose, which may not be captured by more established risk factors such as glycated hemoglobin [29]. We have reported that GI within a MED-HEP is a determinant for both the postprandial glucose response and glycemic variability [30].

Only a few studies have investigated the effects of GI on measures of subjective health and well-being. A cross-sectional study found glycemic load but not GI to be inversely associated with HRQoL in females with overweight or obesity [31]. There are also experimental studies where a high-glycemic load diet has been associated with more depressive symptoms, mood disturbances, and fatigue compared to a low-glycemic load diet [32,33]. However, to our knowledge, there have been no intervention studies investigating the effect of GI on HRQoL in a population with increased cardiometabolic risk. Regarding the effect of GI on sleep health, previously reported findings are inconsistent. Carbohydrate-based meals with high GI ingested 4 h before bedtime have been reported to reduce sleep onset latency (the time it takes to fall asleep) compared to similar meals with low GI in healthy young males [34]. According to cross-sectional results, a high dietary GI was associated with good sleep quality in the general population [35]. Nevertheless, recent longitudinal findings suggest that chronic consumption of carbohydrates with high GI could be a risk factor for insomnia in postmenopausal females [36].

As part of the MEDGICarb-intervention trial [30,37], we aimed to investigate the effect of a low- compared with high-GI MED-HEP on HRQoL and subjective measures of sleep among participants with ≥2 features of the metabolic syndrome. We hypothesized that consumption of a MED-HEP would improve these secondary outcomes with no difference between the groups.

## Methods

The MEDGICarb-intervention trial [30,37] was a 15-wk randomized, controlled, parallel-group multi-center trial conducted at 3 sites: *1*) Federico II University - Naples, Italy *2*) Chalmers University of Technology - Gothenburg, Sweden, and *3*) Purdue University - West Lafayette, IN, United States. The trial, conducted from January 2018 until March 2020, included a 3-wk baseline testing period followed by 12 wk of a controlled dietary intervention. The study protocol was approved by the institutional review board of Federico II University and Purdue University and by the Regional Ethical Review Board of Gothenburg, Sweden. All participants signed an informed consent, and the study complied with the Declaration of Helsinki. The trial is registered in the public trial database clinicaltrials.gov as NCT03410719.

### Experimental design

During the 12-wk intervention period, participants consumed a controlled, eu-energetic, weight-maintenance diet profiled as a MED-HEP with either low GI (experimental) or high GI (control). Subjective measures of HRQoL and sleep were assessed through in-person questionnaires at the baseline testing period, while participants consumed their habitual self-selected diets, and during the last 2 wk of the 12-wk intervention period (postintervention), while participants continued to consume the MED-HEPs. All participants were advised to maintain their habitual types and levels of physical activity during the intervention.

### Participants

The inclusion criteria were established to select middle-aged and older adults at risk for type 2 diabetes mellitus or cardiovascular disease. Adults with ≥2 features of the metabolic syndrome according to the National Cholesterol Education Program’s Adult Treatment Panel III [38] were recruited, of which 1 feature had to be abdominal obesity (waist circumference >102 cm for males or >88 cm for females). The other feature(s) could include elevated blood pressure (>130/85 mmHg or taking medication to control high blood pressure), raised fasting plasma glucose (5.6–7.0 mmol/L), raised fasting triglycerides (1.7–4.5 mmol/L) or reduced HDL cholesterol (<1.0 mmol/L for males or <1.3 mmol/L for females). Additional inclusion criteria were age 30–69 y, BMI 25–37, and stable weight (± 3 kg) during the previous 3 mo. Additional exclusion criteria were: acute illness or cardiovascular events during the previous 6 mo, diabetes, anemia, renal- or liver failure, pregnancy or lactation, a diet incompatible with protocol diets (including vegetarians), smoking *>*20 cigarettes per day or intensive physical activity (≥ 3 h/wk). The participants were randomly assigned to the intervention groups by a research team member at each study site who was not involved in data collection or analysis. The randomization code remained blinded for investigators until all tests and analyses of samples for a priori primary outcomes were finished. Full inclusion criteria, details of recruitment, randomization, and consent procedures are published elsewhere [37].

### Dietary intervention

All participants in both groups were instructed to consume a MED-HEP with the same quantities of metabolizable carbohydrate (270 g/d) and fiber (35 g/d) and sufficient total energy for weight stability. During the 3-wk baseline testing period, all participants consumed their usual, self-selected, unrestricted diets. This was then followed by the 12-wk controlled intervention period, where intervention-specific foods were used to achieve a MED-HEP with either high or low GI. The group-specific diets contained mainly the same foods and beverages, except for exchanges of major sources of starch in the meals, to achieve the difference in GI. For this purpose, one-half of the daily carbohydrate intake (135 g) was different between the 2 intervention groups. Specifically, 135 g of carbohydrates in the low-GI group came from foods with GI values <55 (such as pasta, brown rice, flatbread, and wheat plus rye bread and seeds), whereas 135 g of carbohydrates in the high-GI group came from foods with GI values >70 (such as jasmine rice, potatoes, mashed potatoes, couscous, wholegrain-bread, and rusks). The group-specific carbohydrates were distributed as follows: 35 g at breakfast, 40 g at lunch, and 60 g at dinner. The other half of the daily carbohydrate intake (135 g) was the same for both groups, including carbohydrates in fruits, vegetables, and other foods. Each participant’s total energy intake for weight maintenance was achieved through adjustments of dietary fat and protein.

Participants were provided with intervention-specific food items to consume for their meals as well as with instructions on the quantities of the specific foods during the intervention. All participants were also assisted with prescribed menus for breakfast, lunch, dinner, and snacks together with a “Dinner Recipe Builder.” The purpose of the dinner recipe builder was to enable flexibility to mix and match prescribed foods consumed at dinner while still following the assigned low- or high-GI MED-HEP. Dietary counseling was given bi-weekly, which included cooking classes to help participants cook MED-HEP meals and to build self-efficacy in the ability to follow the dietary intervention. Complete descriptions of dietary adherence are published [37].

### Assessment of HRQoL

To measure HRQoL, the MOS SF-36 version 2 was administrated at baseline and postintervention at all sites. The questionnaire consists of 36 questions that measure 8 domains of subjective health and well-being during the preceding week [[39], [40], [41]]. Data were entered into the program ware Optum ProCoRE (Optum Inc.) for algorithmic transformation into domain scores for the areas of physical functioning, role limitations due to physical health (role physical), bodily pain, general health, vitality, social functioning, role limitations due to emotional health (role emotional) and mental health. The domain scores are presented on a 0–100 scale of arbitrary units (AU), where a higher score indicates greater well-being (0 AU meaning worst possible health state and 100 AU meaning best possible health state). A component summary score was also computed for the aggregated dimensions of physical health and mental health, which are presented as a norm-based T-score (mean = 50 AU, SD = 10 AU).

Scoring of the health domains involves the following steps: *1*) entering response data into the program, *2*) recoding item response choices to values, *3*) calculating total raw scores for each domain by summarizing recoded response values for all items in a given domain, *4*) transformating domain total raw scores to 0–100 scores, *5*) transformating 0–100 scores to *z*-scores for each domain, and *6*) calculating component summary scores for physical health and mental health by using the domain *z*-scores. The component summary scores were computed by *1*) multiplying each domain *z*-score by a specific score coefficient for physical- and mental health, *2*) calculating aggregated component summary scores by summing the resulting products, and *3*) converting the products total to a norm-based T-score for physical- and mental health [42].

### Assessments of sleep

To investigate the dietary effects on subjective sleep quality, the Pittsburgh Sleep Quality Index (PSQI) was administrated at baseline and postintervention at all sites. This is a questionnaire containing 19 self-rated questions that measure sleep quality and sleep disturbances during the preceding month [43]. The result is presented as a global sleep score on a 0–21 AU scale, where a higher score indicates worse sleep quality and a value >5 AU is classified as “poor sleep” [43]. Component scores are also calculated for the areas of subjective sleep quality, sleep latency, sleep duration, habitual sleep efficiency, sleep disturbances, use of sleeping medication, and daytime dysfunction, each on a 0–3 AU scale.

To assess daytime sleepiness, the Epworth Sleepiness Scale (ESS) was distributed at baseline and postintervention at 2 sites (United States and Italy). This is a questionnaire that evaluates the general level of daytime sleepiness through 8 questions [44]. The result is presented as an ESS-score on a 0–24 AU scale, where a higher score indicates greater daytime sleepiness and a value >10 AU is classified as excessive daytime sleepiness [44].

A previous publication describing the study design [37] specified that Actigraphy data would be collected during baseline and postintervention in Sweden and the United States to supplement the questionnaires with objective measures of sleep. These data were, however, not included in the analysis because the quality of the data was not satisfactory.

### Statistical analysis

This is an exploratory analysis of secondary outcomes in the MEDGICarb-intervention trial, and therefore, the statistical analyses were performed on completers of the intervention and without adjustment for multiple testing. For normally distributed data, parametric tests were used, and for skewed data, nonparametric tests were applied. To compare differences between the intervention groups with regard to changes in HRQoL and sleep from baseline to postintervention, a 3-way analysis of variance was conducted. In the models, GI (high or low), site (Italy, United States, and Sweden), and sex were selected as fixed factors, and age and BMI as covariates. Interactions between fixed factors were analyzed and removed if nonsignificant (*P* > 0.1). To investigate the effect from baseline to postintervention, the Wilcoxon signed-rank test was applied for both groups combined as well as for analysis of changes within the groups. Results are presented as least square means ± SE for the analysis of variance models and as mean ± SD for other tests. All statistical analyses were performed with IBM SPSS Statistics 28.0.1.0 software (IBM Corporation), and significance was defined as *P* < 0.05.

## Results

### Baseline characteristics

**FIGURE 1:**
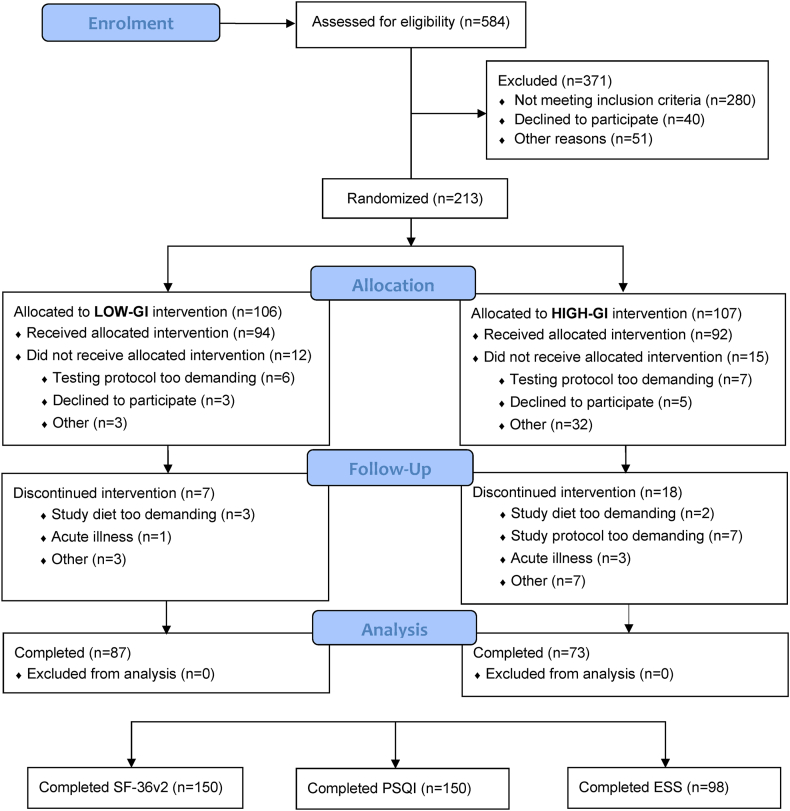
CONSORT flow diagram of participants. CONSORT, consolidated standards of reporting trials; ESS, Epworth Sleepiness Scale; GI, glycemic index; PSQI, Pittsburgh Sleep Quality Index; SF-36v2, Medical Outcomes Study 36-item short-form health survey version 2.

In total, 160 participants completed the dietary intervention (of which 87 were from the low-GI group and 73 from the high-GI group). Of these, 150 participants completed the questionnaires for HRQoL and sleep quality, and 98 participants completed the questionnaire for daytime sleepiness (see CONSORT participant flow diagram in Figure 1). In total, 85 of the participants were females, and 75 were males (mean age 56 ± 10 y, mean BMI 31.0 ± 3.1). At baseline, the completers of the dietary intervention had a subjective health and well-being that was comparable or better than that of the general American population [42] for both the aggregated physical- and mental components of HRQoL (see Table 1 [42]). In terms of sleep, 57 % of the study population was characterized as having poor sleep quality according to PSQI (defined as a global sleep score >5 AU). With regards to daytime sleepiness, 18% of the participants reported having excessive daytime sleepiness (defined as an ESS-score >10 AU). There was no difference between the groups in terms of HRQoL, sleep status, or covariates such as age and BMI at baseline (*P* > 0.05, Table 1 [42]).

**TABLE 1:** Baseline characteristics of completers of the dietary intervention presented as means ± SD

Variable^1^	All (*n* = 160)	High-GI diet (*n* = 73)	Low-GI diet (*n* = 87)
Age, y	56 ± 10	56 ± 10	56 ± 11
Female, *n* (%)	85 (53)	37 (51)	48 (55)
BMI, kg/m^2^	31.0 ± 3.1	30.9 ± 2.9	31.0 ± 3.2
Physical functioning^2^, AU	86.1 ± 16.3	86.7 ± 16.5	85.6 ± 16.3
Role physical^2^, AU	87.2 ± 20.6	89.2 ± 16.8	85.4 ± 23.3
Bodily pain^2^, AU	75.2 ± 24.6	72.5 ± 24.1	77.5 ± 24.9
General health^2^, AU	68.1 ± 20.2	64.9 ± 22.6	70.9 ± 17.6
Vitality^2^, AU	62.1 ± 19.9	61.3 ± 19.2	62.8 ± 20.6
Social functioning^2^, AU	85.0 ± 19.3	83.2 ± 20.1	86.6 ± 18.7
Role emotional^2^, AU	90.6 ± 15.7	88.9 ± 15.8	92.0 ± 15.6
Mental health^2^, AU	77.5 ± 16.0	75.3 ± 17.0	79.4 ± 14.9
Physical component summary^3^, AU	52.0 ± 7.4	52.1 ± 7.4	52.0 ± 7.5
Mental component summary^3^, AU	51.2 ± 8.0	50.1 ± 8.4	52.2 ± 7.6
Global sleep score^4^, AU	6.5 ± 3.2	6.7 ± 2.9	6.4 ± 3.4
Daytime sleepiness^5^, AU	6.6 ± 4.1	6.6 ± 4.2	6.6 ± 4.0

Abbreviations: AU, arbitrary unit; BMI, body mass index; GI, glycemic index; HRQoL, health-related quality of life; PSQI, Pittsburgh Sleep Quality Index; SD, standard deviation; SF-36v2, Medical Outcomes Study 36-item short-form health survey version 2.

1 There was no difference between the groups in terms of age, BMI, HRQoL, or sleep status at baseline (age and BMI tested with independent t-test and study outcomes with Mann-Whitney test, P > 0.05).

2 Measured with SF-36v2 (raw scores, scale 0–100).

3 Measured with SF-36v2. Means are presented as norm-based T-scores; a score ≥47 is comparable or better than that of the general American population [42].

4 Measured with PSQI (scale 0–21, a scoring >5 indicates poor sleep).

5 Measured with Epworth Sleepiness Scale (scale 0–24, a scoring >10 indicates excessive daytime sleepiness).

### Dietary intake

Compared to baseline, both groups increased their intakes of energy (kcal/d), protein [energy percentage (% E)], carbohydrates (g/d), and fiber (g/d), whereas their intakes of fat (% E), saturated fat (% E), polyunsaturated fat (% E) and alcohol (g/d) decreased (*P* < 0.05, Table 2). Postintervention, targeted differences in GI between the groups were achieved with a mean GI value of 46.7 ± 7.6 for low GI and 62.8 ± 9.6 for high GI (*P* < 0.001). Otherwise, there were no differences in energy intake or nutrient composition between the low- and high-GI groups at baseline or postintervention (*P* > 0.05, Table 2).

**TABLE 2:** Dietary composition at baseline and after the dietary intervention presented as means ± SD

	Baseline^1^		Postintervention^2^	
	High-GI (*n* = 65)	Low-GI (*n* = 78)	High-GI (*n* = 65)	Low-GI (*n* = 78)
Energy (kcal/d)	1937 ± 506	1924 ± 504	2250 ± 442∗∗	2338 ± 610∗∗
Protein (% E)	16.6 ± 3.1	16.7 ± 3.0	18.6 ± 2.8∗∗	18.6 ± 3.6∗∗
Fat (% E)	38.2 ± 5.9	38.3 ± 6.2	35.8 ± 5.6∗	35.8 ± 6.3∗
SFA (% E)	13.5 ± 4.0	13.1 ± 3.9	10.0 ± 3.2∗∗	9.7 ± 3.0∗∗
MUFA (% E)	14.6 ± 3.2	15.1 ± 3.4	16.5 ± 3.0∗	16.0 ± 4.0
PUFA (% E)	5.7 ± 2.6	5.7 ± 2.5	4.8 ± 1.4∗	4.7 ± 1.6∗∗
Cholesterol (mg/d)	294 ± 131	291 ± 144	340 ± 212	330 ± 213
Carbohydrates (g/d)	221.5 ± 62.5	220.0 ± 67.0	251.8 ± 35.8∗∗	260.0 ± 45.0∗∗
Carbohydrates (% E)	43.0 ± 5.9	42.9 ± 6.5	42.8 ± 6.4	42.9 ± 6.5
Sugars (% E)	13.8 ± 5.7	13.0 ± 5.3	12.4 ± 7.2	11.5 ± 6.2∗
Fiber (g/d)	20.5 ± 6.0	19.5 ± 7.6	30.8 ± 5.8∗∗	32.1 ± 6.8∗∗
GI (AU)	59.2 ± 9.2	59.0 ± 10.0	62.8 ± 9.6∗	46.7 ± 7.6∗∗
Alcohol (g/d)	7.8 ± 10.7	10.0 ± 14.0	3.5 ± 5.5∗∗	2.8 ± 6.3∗∗

Abbreviations: AU, arbitrary unit; GI, glycemic index; MUFA, monounsaturated fatty acid; PUFA, polyunsaturated fatty acid; SD, standard deviation; SFA, saturated fatty acid; % E, energy percentage.

1 There was no difference in energy or nutrient composition between the low- and high-GI at baseline (*P* > 0.05).

2 Postintervention targeted differences in GI between groups were achieved (*P* < 0.001); otherwise, there was no difference in energy or nutrient composition between the low- and high-GI (*P* > 0.05).

∗ Significant difference between baseline and postintervention, *P* < 0.05 (paired t-test).

∗∗ Significant difference between baseline and postintervention, *P* < 0.001 (paired t-test).

### HRQoL

**FIGURE 2:**
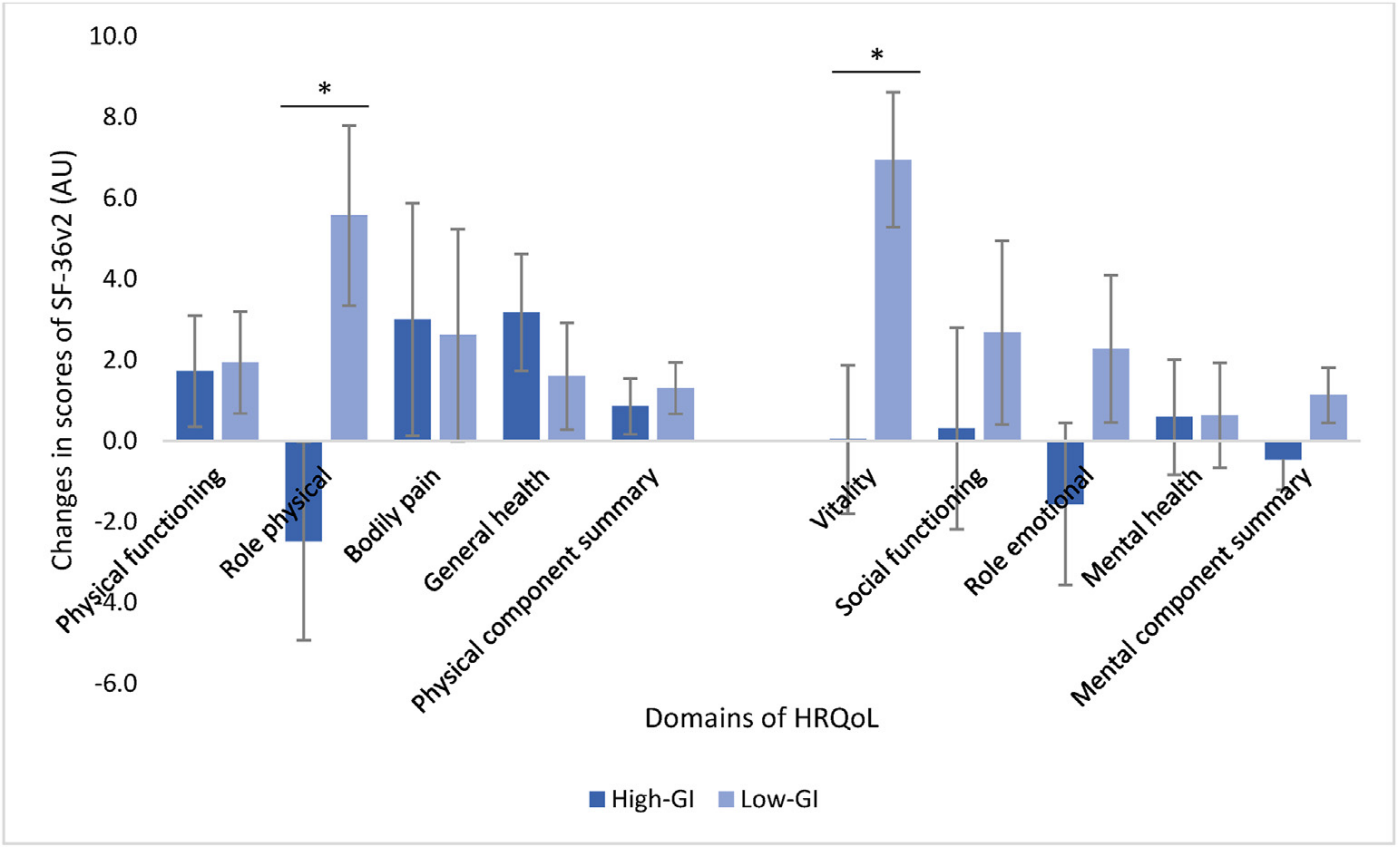
Changes in scores of self-perceived HRQoL (SF-36v2) after consuming a MED-HEP with either high or low GI foods for 12 wk (*n* = 150). Values are presented as LSM ± SE extracted from a 3-way ANOVA with the intervention group, site, and sex as fixed factors adjusted for age and BMI. ∗Significant difference between groups, *P* < 0.05. ANOVA, analysis of variance; BMI, body mass index; GI, glycemic index; HRQoL, health-related quality of life; LSM, least square means; MED-HEP, Mediterranean healthy eating pattern; SE, standard error; SF-36v2, Medical Outcomes Study 36-item short-form health survey version 2.

Consuming the MED-HEPs resulted in differential changes between the groups in domains of HRQoL from baseline to postintervention (Figure 2). The changes were mostly driven by improvements in the low-GI group, where the health domains of role physical (5.6 ± 2.2 AU for low-GI compared with –2.5 ± 2.5 AU for high-GI) and vitality (6.9 ± 1.7 AU for low-GI compared with 0.0 ± 1.8 AU for high-GI) were different (*P* < 0.05). No differences were found between the 2 diets for changes in aggregated physical- or mental components or for the other domains of HRQoL (physical functioning, bodily pain, general health, social functioning, role emotional, and mental health). For vitality, there was a significant effect of age, which was also seen for the aggregated mental components (*P* < 0.05). A post hoc correlation analysis demonstrated a modest positive association between age and scoring of the aggregated mental components of HRQoL (r_s_ = 0.36, *P* < 0.001) at baseline and an inverse relationship between age and change in aggregated mental components of HRQoL (r_s_ = –0.20, *P* < 0.05).

For both groups combined, there were significant improvements from baseline to postintervention for the domains of general health (68.9 ± 19.4 AU compared with 71.1 ± 17.3 AU), vitality (62.8 ± 19.2 AU compared with 66.5 ± 17.8 AU) as well as the physical component summary (52.2 ± 7.2 AU compared with 53.3 ± 7.2 AU), *P* < 0.05. The improvements for both groups combined were mainly driven by improvements within the different intervention groups, where similar patterns were seen (high-GI; general health 66.4 ± 21.1 AU compared with 69.4 ± 17.1 AU, *P* < 0.05), low-GI; role physical 85.6 ± 23.4 AU compared with 90.9 ± 20.1 AU, *P* < 0.05; vitality 62.9 ± 20.7 AU compared with 69.7 ± 18.0 AU, *P* < 0.001). For values at baseline, postintervention, and changes from baseline to postintervention (displayed as means ± SD), see Supplemental Table 1.

### Measures of sleep

**FIGURE 3:**
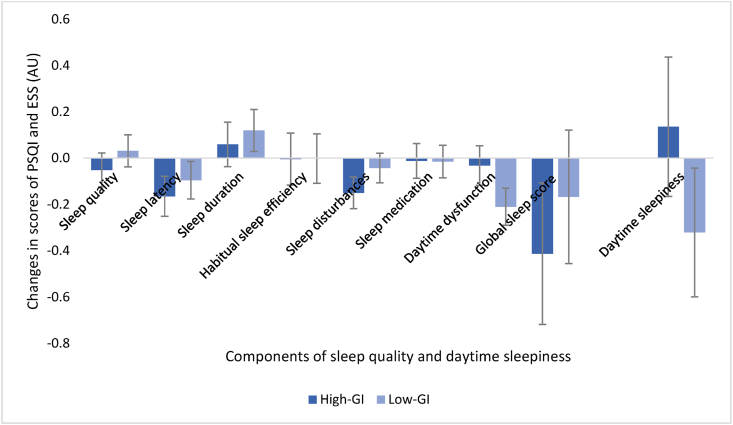
Changes in scores of subjective sleep quality (PSQI, *n* = 150) and daytime sleepiness (ESS, *n* = 98) after consuming a MED-HEP with either high or low GI for 12 wk. Values are presented as least LSM ± SE extracted from a 3-way ANOVA with the intervention group, site, and sex as fixed factors adjusted for age and BMI. There were no significant differences between the groups, *P* > 0.05. ANOVA, analysis of variance; BMI, body mass index; ESS, Epworth Sleepiness Scale; GI, glycemic index; LSM, least square means; MED-HEP, Mediterranean healthy eating pattern; PSQI, Pittsburgh Sleep Quality Index; SE, standard error.

There were no differences between the groups for changes in component scores or global sleep score of sleep quality (PSQI) from baseline to postintervention (Figure 3). For both groups combined, improvements were seen in the components scores sleep latency (1.1 ± 1.0 AU at baseline compared with 0.9 ± 0.9 AU postintervention, corresponding to a 7% improvement on the 0–3 sleep scale) and daytime dysfunction (0.7 ± 0.7 AU at baseline compared with 0.6 ± 0.7 AU postintervention, corresponding to a 3% improvement), *P* < 0.05. As for HRQoL, the changes for both groups combined were driven by improvements in the different intervention groups (high GI; sleep latency 1.0 ± 0.9 AU compared with 0.9 ± 0.9 AU; sleep disturbances 1.4 ± 0.5 AU compared with 1.3 ± 0.6 AU, low-GI; daytime dysfunction, 0.7 ± 0.7 AU compared with 0.5 ± 0.6 AU, *P* < 0.05). For changes in daytime sleepiness (ESS), there was no significant difference in effect between groups. Neither was the change from baseline to postintervention for both groups combined or within the groups. For values at baseline, postintervention, and changes from baseline to postintervention for measures of sleep (displayed as means ± SD), see Supplemental Table 1.

## Discussion

To the best of our knowledge, the current study is the first reported to investigate the effect of GI on HRQoL in populations with increased cardiometabolic risk. In the current exploratory analysis of the MEDGICarb-intervention trial, we found that low compared to high GI in the context of a MED-HEP resulted in modest improvements in some domains of HRQoL, whereas no major differences were seen between the groups for indexes of sleep.

The effect of a MED-HEP on HRQoL has previously been investigated in small intervention studies, where improvements have been demonstrated in specific domains of HRQoL. In a 5-wk cross-over-trial, the influence of a MED-HEP with different amounts of red meat on subjective health and well-being was studied in adults with overweight or obesity [45]. Similar effects were seen in the health domains role physical and vitality as in our study, irrespective of red meat content. In another intervention study [46], the effect of a hypocaloric MED-HEP with and without the addition of moderate-to-high-intensity endurance training was investigated in a population with metabolic syndrome. Improvements were reported in the domains of physical function, general health, vitality, and role emotional after 12 wk of a MED-HEP. The effects were further enhanced with the addition of endurance training. As the intervention resulted in weight loss, it is however, likely that the effect was at least partly related to the reduction in weight.

In our study, the changes in the health domains’ role, physical, and vitality were different between the low- compared to high-GI groups, but the differences were modest, and it remains to be understood to what extent they are of practical meaning. Within the low-GI group, these domains improved from baseline to postintervention, whereas the high-GI group showed no changes in these components. In contrast, a significant within-group improvement was seen in the domain of general health for the high-GI group but not for the low-GI group. For the domain vitality as well as the aggregated mental components, significant effects were found from age. A post hoc correlation analysis demonstrated an inverse relationship between age and change in mental dimensions, suggesting a better treatment effect for younger participants for the mental components of HRQoL. Previous research has indicated a decreasing score with age for the aggregated physical components and an inverse relationship for the aggregated mental components in a population with metabolic syndrome [7]. The latter also applied to this study, which might be a possible explanation for the effects of age. As mental illness is most common among young adults [47], this might propose an important target group for dietary interventions with the aim of improving subjective well-being, where a low GI could be a central component of a HEP. Important to bear in mind is also that the baseline scores were considered similar or better compared to a general population both for the aggregated physical- and mental components, indicating that there could be more room for improvement in a population with a reduced HRQoL.

With regards to sleep, a previous study found that high-GI meals led to a reduced time to sleep onset than low-GI meals [34]. In our study, we found no major differences between the groups in terms of changes in sleep quality or daytime sleepiness during the intervention. Within the high-GI group, however, there was a significant improvement in the components of sleep latency and sleep disturbances from baseline to postintervention, whereas the low-GI group showed a significant improvement in daytime function. It is interesting that both features of GI might have an impact on different aspects of sleep quality, and a natural next step would be to study the influence of GI on objective measures of such parameters. Recent findings have indicated that the quality of the diet might be of importance for the sleep microstructure [48], even if GI was not in particular focus here. In this study, a diet high in sugar and fat led to unfavorable short-term changes in oscillatory features of sleep compared to a healthier diet, which could impact the restorative function of sleep and, thereby, the daily function.

Strengths of the MEDGICarb-intervention trial include the strong study design with a randomized, controlled, parallel-group multi-center trial, where the results were blinded for investigators until analyses of primary outcomes were finalized. There was a robust difference in GI between the 2 intervention groups, which were similar in other dietary aspects. The good quality of the 2 intervention diets is also a strength to enable separation of the effects of GI from other components of the diet. Another strength of the study is the focus on weight maintenance through the intervention, as weight change is a factor with the potential to impact both HRQoL and sleep. Furthermore, there was an equal distribution between males and females among the participants in the study, of which age covered a broad span of an adult population. There are also limitations of the study that must be acknowledged. Firstly, almost all participants were of Caucasian ethnicity, which limits the generalizability of the results to populations with other ethnic backgrounds. Due to the nature of the study, it was not possible to blind the participants to their intervention group assignment. Participants had been given information about the aim of the study before enrolment, and even if the specific groups were not announced during the intervention, information about GI can still be found elsewhere. There was a fairly long run in period for the study, which was necessary to cover all measurements needed for the total study design (not presented in this article). Another limitation of this analysis of secondary outcomes of the intervention is that the power calculation was based on the primary outcomes and not with the measures of HRQoL and sleep in mind, and no adjustments were made for multiple tests.

In conclusion, low compared to high GI resulted in modest improvements in the health domains role physical and vitality, but not in the aggregated physical or mental components or for the other domains of HRQoL (physical functioning, bodily pain, general health, social functioning, role emotional, and mental health) or for sleep quality or daytime sleepiness in the context of a MED-HEP and a population at high risk for developing cardiovascular disease and type 2 diabetes mellitus. This suggests that consuming carbohydrate foods with low GI as part of a MED-HEP may promote additional benefits with regard to some aspects of self-perceived health.

## Acknowledgments

We thank Barilla G. e R. Fratelli S.p.A, Parma, Italy, for providing some of the cereal products for the study participants.

## Author contributions

The authors’ responsibilities were as follows – REB, RL, GR, WWC: designed the research; REB, RG, GC, MV: conducted the research; AH: analyzed the data and wrote the article; RL: had the primary responsibility for the final content; and all authors: read and approved the final manuscript.

## Conflict of interest

GR, WWC, and RL served as co-principal investigators and are thus co-senior authors. GR is a member of the Health and Wellbeing Advisory Board of the Barilla company; remuneration for this activity goes to his University Department. RL is the project leader for the Nordic Rye Forum (www.nordicryeforum.org), for which funding is provided by industrial partners and NKJ (The Nordic Joint Committee for Agricultural and Food Research). RL is also a principal investigator in research projects funded by Lantmännen and Barilla. He did not receive any remuneration, salary, or any other financial recompense from the food industry. REB is currently employed by ADM. The research presented in this article was conducted in a former role and has no connection with ADM. AH is offering nutrition-related services through her own practice as a nutritionist. All other authors report no conflicts of interest.

## Funding

This study was funded by Barilla International and Barilla United States. The funding sources had no role in the collection, analysis, and interpretation of data, in writing this or any other reports, and in the decision to submit the article for publication.

## Data availability

Data described in the manuscript will be made available upon request, pending from the corresponding author.
