# Supplementary material for: Low- versus High-Glycemic Index Mediterranean-Style Eating Patterns Improved Some Domains of Health-Related Quality of Life but Not Sleep in Adults at Risk for Type 2 Diabetes: The MEDGICarb Randomized Controlled Trial
Source: J Nutr. 2024 Jul 14;154(9):2743–51. doi: 10.1016/j.tjnut.2024.07.005 (PMC11393163; doi:10.1016/j.tjnut.2024.07.005)
Supplement: multimedia component [file mmc1.docx]

**Low- vs high-glycemic index Mediterranean-style eating patterns improved some domains of health-related quality of life but not sleep in adults at risk for type 2 diabetes: The MEDGICarb randomized controlled trial, Anna Hjort**

**Supplementary table 1.** Values for completers of the questionnaires at baseline, post-intervention and change from baseline to post-intervention presented as means ± SD.

|  | **High-GI** | | | **Low-GI** | | | **All** | | |
| --- | --- | --- | --- | --- | --- | --- | --- | --- | --- |
|  | **Baseline** | **Post** | **Change** | **Baseline** | **Post** | **Change** | **Baseline** | **Post** | **Change** |
| **SF-36v2** | (n = 68) | | | (n = 82) | | | (n = 150) | | |
| Physical functioning | 87.4 ± 14.8 | 89.0 ± 12.7 | 1.5 ± 9.5 | 86.2 ± 15.6 | 88.2 ± 15.3 | 2.0 ± 12.6 | 86.7 ± 15.2 | 88.5 ± 14.1 | 1.8 ± 11.3 |
| Role physical | 90.3 ± 13.6 | 87.8 ± 17.3 | -2.6 ± 15.6 | 85.6 ± 23.4 | 90.9 ± 20.1 | 5.4 ± 23.1 | 87.7 ± 19.7 | 89.5 ± 18.9 | 1.8 ± 20.4 |
| Bodily pain | 73.4 ± 23.7 | 76.2 ± 23.0 | 2.8 ± 22.9 | 77.7 ± 25.0 | 80.5 ± 23.5 | 2.9 ± 23.7 | 75.7 ± 24.4 | 78.6 ± 23.3 | 2.8 ± 23.3 |
| General health | 66.4 ± 21.1 | 69.4 ± 17.1 | 3.0 ± 12.6 | 71.0 ± 17.6 | 72.5 ± 17.4 | 1.5 ± 11.2 | 68.9 ± 19.4 | 71.1 ± 17.3 | 2.2 ± 11.8 |
| Vitality | 62.8 ± 17.5 | 62.5 ± 16.9 | -0.2 ± 16.3 | 62.9 ± 20.7 | 69.7 ± 18.0 | 6.9 ± 14.8 | 62.8 ± 19.2 | 66.5 ± 17.8 | 3.6 ± 15.8 |
| Social functioning | 84.4 ± 18.9 | 84.4 ± 19.4 | 0.0 ± 18.5 | 86.4 ± 18.8 | 88.9 ± 17.3 | 2.4 ± 22.1 | 85.5 ± 18.8 | 86.8 ± 18.4 | 1.3 ± 20.5 |
| Role emotional | 90.1 ± 14.3 | 88.1 ± 16.2 | -2.0 ± 17.9 | 91.9 ± 15.7 | 94.2 ± 11.2 | 2.3 ± 15.3 | 91.1 ± 15.0 | 91.4 ± 14.0 | 0.4 ± 16.6 |
| Mental health | 76.8 ± 14.8 | 76.7 ± 14.5 | -0.1 ± 12.2 | 79.5 ± 14.9 | 80.3 ± 13.4 | 0.8 ± 12.1 | 78.3 ± 14.9 | 78.7 ± 14.0 | 0.4 ± 12.1 |
| Physical component summary | 52.3 ± 7.0 | 53.1 ± 6.7 | 0.8 ± 4.8 | 52.1 ± 7.4 | 53.4 ± 7.6 | 1.3 ± 6.3 | 52.2 ± 7.2 | 53.3 ± 7.2 | 1.1 ± 5.7 |
| Mental component summary | 50.8 ± 7.4 | 50.2 ± 7.4 | -0.6 ± 7.1 | 52.1 ± 7.6 | 53.2 ± 6.2 | 1.1 ± 5.7 | 51.5 ± 7.5 | 51.8 ± 6.9 | 0.3 ± 6.4 |
| **PSQI** | (n = 70) | | | (n = 80) | | | (n = 150) | | |
| Sleep quality | 1.0 ± 0.7 | 1.0 ± 0.7 | 0.0 ± 0.6 | 1.0 ± 0.7 | 1.1 ± 0.8 | 0.0 ± 0.7 | 1.1 ± 0.6 | 1.0 ± 0.7 | 0.0 ± 0.6 |
| Sleep latency | 1.1 ± 1.0 | 0.9 ± 0.9 | -0.2 ± 0.7 | 1.1 ± 1.1 | 1.0 ± 0.9 | -0.1 ± 0.7 | 1.1 ± 1.0 | 0.9 ± 0.9 | -0.1 ± 0.7 |
| Sleep duration | 1.2 ± 0.9 | 1.3 ± 0.9 | 0.1 ± 0.7 | 1.2 ± 0.9 | 1.4 ± 0.9 | 0.1 ± 0.9 | 1.2 ± 0.9 | 1.3 ± 0.9 | 0.1 ± 0.8 |
| Habitual sleep efficiency | 0.7 ± 1.0 | 0.7 ± 0.9 | 0.0 ± 0.9 | 0.7 ± 1.0 | 0.7 ± 1.1 | 0.0 ± 1.0 | 0.7 ± 1.0 | 0.7 ± 1.0 | 0.0 ± 1.0 |
| Sleep disturbances | 1.3 ± 0.6 | 1.3 ± 0.6 | -0.1 ± 0.6 | 1.3 ± 0.6 | 1.3 ± 0.6 | 0.0 ± 0.6 | 1.4 ± 0.6 | 1.3 ± 0.6 | -0.1 ± 0.6 |
| Sleep medication | 0.3 ± 0.8 | 0.3 ± 0.8 | 0.0 ± 0.5 | 0.3 ± 0.8 | 0.3 ± 0.8 | 0.0 ± 0.7 | 0.3 ± 0.8 | 0.3 ± 0.8 | 0.0 ± 0.6 |
| Daytime dysfunction | 0.7 ± 0.7 | 0.7 ± 0.8 | 0.0 ± 0.7 | 0.7 ± 0.7 | 0.5 ± 0.6 | -0.2 ± 0.7 | 0.7 ± 0.7 | 0.6 ± 0.7 | -0.1 ± 0.7 |
| Global sleep score | 6.4 ± 3.5 | 6.2 ± 3.2 | -0.3 ± 2.5 | 6.4 ± 3.5 | 6.3 ± 3.9 | -0.1 ± 2.7 | 6.5 ± 3.2 | 6.3 ± 3.6 | -0.2 ± 2.6 |
| **ESS** | (n = 45) | | | (n = 53) | | | (n = 98) | | |
| Daytime sleepiness | 6.4 ± 4.2 | 6.5 ± 3.9 | 0.1 ± 2.4 | 6.5 ± 4.0 | 6.2 ± 4.2 | -0.3 ± 1.7 | 6.5 ± 4.1 | 6.4 ± 4.1 | -0.1 ± 2.0 |

Abbreviations: ESS, Epworth Sleepiness Scale; GI, Glycemic Index; PSQI, Pittsburgh Sleep Quality Index, SD, Standard Deviation; SF-36v2, Medical Outcomes Study 36-Item Short Form Health Survey Version 2.
